# Using Bayesian networks to discover relations between genes, environment, and disease

**DOI:** 10.1186/1756-0381-6-6

**Published:** 2013-03-21

**Authors:** Chengwei Su, Angeline Andrew, Margaret R Karagas, Mark E Borsuk

**Affiliations:** 1 Thayer School of Engineering, Dartmouth College, Hanover, NH, USA; 2 Community and Family Medicine, Section of Biostatistics & Epidemiology, Geisel School of Medicine, Hanover, NH, USA

**Keywords:** Structural learning, Belief networks, Genetic epidemiology, Bioinformatics, Complex traits, Arsenic, SNP

## Abstract

We review the applicability of Bayesian networks (BNs) for discovering relations between genes, environment, and disease. By translating probabilistic dependencies among variables into graphical models and vice versa, BNs provide a comprehensible and modular framework for representing complex systems. We first describe the Bayesian network approach and its applicability to understanding the genetic and environmental basis of disease. We then describe a variety of algorithms for learning the structure of a network from observational data. Because of their relevance to real-world applications, the topics of missing data and causal interpretation are emphasized. The BN approach is then exemplified through application to data from a population-based study of bladder cancer in New Hampshire, USA. For didactical purposes, we intentionally keep this example simple. When applied to complete data records, we find only minor differences in the performance and results of different algorithms. Subsequent incorporation of partial records through application of the EM algorithm gives us greater power to detect relations. Allowing for network structures that depart from a strict causal interpretation also enhances our ability to discover complex associations including gene-gene (epistasis) and gene-environment interactions. While BNs are already powerful tools for the genetic dissection of disease and generation of prognostic models, there remain some conceptual and computational challenges. These include the proper handling of continuous variables and unmeasured factors, the explicit incorporation of prior knowledge, and the evaluation and communication of the robustness of substantive conclusions to alternative assumptions and data manifestations.

## Introduction

It is now widely understood that most diseases have a genetic component and yet do not follow the simple Mendelian inheritance patterns of dominant or recessive traits [1]. Presumably, these diseases result from the interacting effects of multiple genes in combination with one or more environmental risk factors [2]. Such complex phenotypic traits are characterized by a high level of unpredictability, as both the number and nature of interactions are difficult to distinguish using conventional methods. Nevertheless, uncovering the genetic basis for disease and deciphering the relative contribution of environmental exposure are critical steps toward the goal of developing an effective system of “personalized” medicine [3,4].

Machine learning methods, in particular Bayesian networks (BNs), have the potential to help disentangle the web of relations among genes, environment, and disease [5]. BNs are a multivariate modelling method able to simultaneously account for gene-gene (epistasis) and gene-environment interactions, as well as leverage the diagnostic potential of clinical or physiological factors [6]. BNs also lead directly to prognostic models: a constructed network, however complex, can be used to efficiently compute the probability that an individual with a particular genotype and environmental exposure will exhibit the phenotype of interest. BNs have been applied in a variety of settings for the purposes of causal study and probabilistic prediction, including medical diagnosis, crime and terrorism risk, forensic science, and ecological conservation (see [7]). In bioinformatics, they have been used to analyze gene expression data [8,9], derive protein signaling networks [10-12], predict protein-protein interactions [13], perform pedigree analysis [14], conduct genetic epidemiological studies [5], and assess the performance of microsatellite markers on cancer recurrence [15]. In this paper, we review the potential for BNs to contribute to revealing the genetic and environmental basis of disease.

## Bayesian networks

A Bayesian network is a graphical model of the relationships among a set of random variables. It consists of two components:

a) A network *structure* in the form of a directed acyclic graph (DAG). In this graph, nodes represent the random variables and directed edges represent stochastic dependencies among variables.

b) A set of conditional probability distributions, one for each variable, characterizing the stochastic dependencies represented by the edges. These conditional distributions are specified by the network *parameters*[16].

If there is a directed edge in a DAG from node Y to node Z, Y is said to be a *parent* of Z; likewise Z is called a *child* of Y. An important feature of a BN is that each variable represented by a node is understood to be conditionally independent of the set of all its predecessors in the graph, given the values of its parents [17]. In other words, the absence of a directly connecting arrow between any two nodes implies that these two variables are independent given the values of any intermediate nodes. This is referred to as the *Markov condition*. Based on the Markov condition, the joint probability distribution for the entire set of variables represented by a BN can be decomposed into a product of conditional probabilities using the graphical structure and the chain rule of probability calculus:

(1) $$p\left(x\left|\theta \right)\right)=\prod_{i=1}^{n}p\left(x_{i}\left|\mathrm{pa}\left(x_{i}\right),\theta_{i}\right)\right)$$

where ***x*** = {*x*_*1*_*,…x*_*n*_} are the variables (nodes in the BN) and **θ** = {*θ*_*1*_*,…,θ*_*n*_} are the BN’s parameters, where each *θ*_*i*_ is the set of parameters necessary to specify the distribution of the variable *x*_*i*_ given its parents **pa**(*x*_*i*_). (We adopt the notation of [18] in which lower case symbols are used to indicate particular realizations of the corresponding uppercase variables.) Importantly, the factorization given by eq. (1) allows complex systems to be analyzed and modeled using a limited number of local relationships as building blocks.

**Figure 1:**
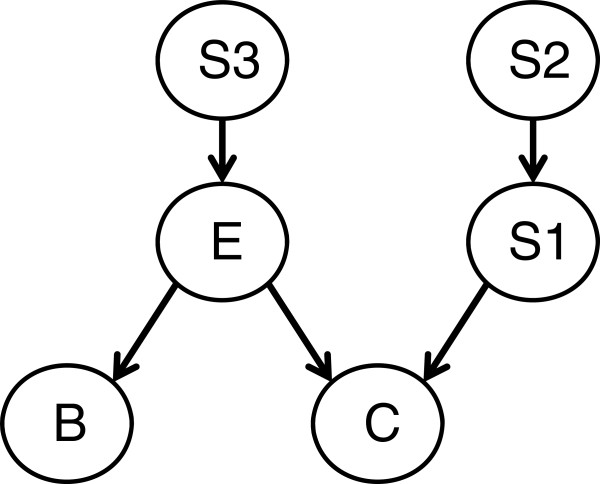
**A simple BN representing the relationship between cancer incidence (C), environmental exposure (E), a biomarker (B) and three single nucleotide polymorphisms (S1, S2, S3).** See text for further description.

Figure 1 illustrates a simple hypothetical BN relating the incidence of cancer (C) to an environmental exposure (E), a biomarker of exposure (B) and three single nucleotide polymorphisms (SNPs). (We assume that there are no latent or hidden variables in this particular BN.) There is an edge shown from E to C, indicating that cancer risk is understood to be dependent on environmental exposure. There is also an edge from S1 to C indicating a genetic influence on cancer susceptibility, either directly or through mediation of the exposure effect. The node S2 represents an older SNP that may be associated with S1 through evolution. These two SNPs do not necessarily need to be on the same gene or in the same region of linkage disequilibrium [19]. Additionally, E is shown to be dependent on S3, indicating that there is a genetic component to exposure (a genetic predisposition to smoking, for example). Finally, E is a parent of B, reflecting B’s role as a diagnostic biomarker of exposure. Importantly, in this graph there is no directed edge from either node S2 or S3 to C, nor from S3 to B. This implies that knowing the states of E and S1 renders C and B independent of S3 and S2. The joint distribution of all five variables can thus be factored according to equation [1] as:

(2) $$P\left(S1,S2,S3,E,B,C\right)=P\left(B\left|E\right)\right)\cdot P\left(C\left|E,S1\right)\right)\cdot P\left(E\left|S3\right)\right)\cdot P\left(S1\left|S2\right)\right)\cdot P\left(S3\right)\cdot P\left(S2\right)$$

If each of the five variables in Figure 1 is assumed to be binary, this factorization reduces the number of parameters (conditional probabilities) required to specify the full joint distribution from 64 to 12. This allows large networks to be parameterized from relatively small data sets while still capturing the kinds of interactions that are typical of complex traits.

## Genetic dissection of disease

A number of recent reviews [20-22] emphasize the complex nature of the gene-environment-disease relationship and call for new statistical methods to address such complexities. The parametric linear modeling framework (e.g. logistic regression) traditionally used in association studies, while familiar and easy to implement and interpret, is limited in its ability to detect interactions [23]. Studies employing this approach also typically start by considering only one SNP at a time and then include interaction effects only for those SNPs that exhibit independent marginal effects [22]. This procedure ignores the broader environmental context and multigenic nature of complex disease. It also runs the risk of identifying too many associated SNPs as a result of linkage disequilibrium or evolutionarily induced dependencies.

These concerns are exemplified by the relationships shown in Figure 1. A simple one-at-a-time search procedure might identify associations between C, E, and S1, as long as the environmental exposure and SNP both have direct effects on cancer. But if S1 serves primarily to mediate the effects of E on C, its role would be missed if interactions are not properly considered. Further, because of its association with S1, it is possible that S2 would be identified as being associated with C. This would introduce a redundancy if S2 and S1 are in linkage disequilibrium, or a false association if S2 and S1 are not on the same gene or region of linkage disequilibrium [19]. Finally, traditional approaches focused exclusively on direct relations would likely overlook the roles of S3 and B, although their identification could have utility for disease prediction, identification, and prevention.

Improved procedures for implementing linear models can partially address these shortcomings by putting more emphasis on interactions [24] or efficient parameterizations [25]. Yet, the high numbers of higher-order interactions that are now believed to underlie most human disease are not compatible with the strengths of traditional statistical methods [26]. Multifactor dimensionality reduction (MDR) is one example of a novel computational strategy for detecting and characterizing multiple non-linear interactions in the absence of detectable marginal effects [27,28]. MDR works by pooling genotypes from multiple loci to create new variables before subsequent associative analysis. A user-friendly open source software package for implementing MDR is freely available from http://www.epistasis.org.

Even after pre-processing by MDR, linear logistic regression-type methods require large sample sizes to achieve significant power. They also require focus on a single well-defined phenotype. In contrast, through efficient factorization, BNs can break down the discovery process of a complex system into separate investigations of smaller components [29]. This means disease phenotypes can be defined by multiple factors, including clinical or physiological diagnostic variables. Further, as opposed to creating problems of multicollinearity, the associations between candidate predictor variables are naturally accounted for when defining a BN’s conditional probability distributions [19]. In the next section, we describe the means by which BNs can be used in genetic association studies.

## Structural learning

*Learning* a BN can refer to data-based inference of either: (i) the conditional probability parameters for a given structure or (ii) the underlying graphical structure itself. We focus here on the process of *structural learning*, as the discovery of novel relations between genes, the environment, and disease is typically considered a harder problem of greater interest than the estimation of effect strength for a known factor. Often, parameter values are estimated concurrently as part of structural learning.

### Methodological approaches

Many techniques have been developed to learn the structure of BNs from data. Generally, the available algorithms can be classified into two types of approaches, *constraint-based* and *score-based*:where $p\left(D\left|\hat{\theta }\right),G\right)$ is the likelihood of the data D according to estimated parameters $\hat{\theta }$ and structure G, N is the sample size of the dataset, and *n*_*p*_ is the number of parameters. The second term serves to penalize networks with many edges, thus the BIC will lead to a preference for simpler graphs. For large N, the highest scoring model often has parameters that are close to the maximum likelihood values.

•***Constraint-based methods*** focus on identifying conditional independence relationships (i.e., Markov conditions) between variables using observed data. These conditional independencies can then be used to constrain the underlying network structure. This approach was pioneered by Glymour et al. [30], Spirtes et al. [31], and Verma and Pearl [32]. Typically, hypothesis testing procedures, such as the *χ*^2^ test, are first used to remove edges from a fully connected undirected graph based on findings of unconditional independence. Next, directions are added to edges between nodes according to the *d-separation* criterion (short for *directed* separation). If X, Y, Z are three disjoint sets of nodes in a BN, then Y is said to *d-separate* X from Z if and only if Y blocks every path from a node in X to a node in Z [18]. In Figure 1, node E d-separates both B and C from S3, and S1 d-separates C from S2 (all *serial* paths). As part of a *divergent* path, E also d-separates nodes B and C from each other. Importantly, however, node C does *not* d-separate E from S1 because *convergent* paths are *not* blocked when conditioning upon the node at the convergence point or its descendants.

•All d-separation relations between nodes in a graph imply conditional independence relations between the corresponding variables. The difference between the probabilistic dependencies implied by serial, divergent, and convergent paths, therefore, is essential to inferring edge direction from statistical analysis of data. Therefore, if the graph in Figure 1 represents the true underlying dependence structure, we would expect to find the corresponding conditional independencies in the data. However, it is not always the case that a particular set of conditional independencies specify a unique directed graph. Alternatively, the power of a particular data set to identify conditional independencies may be limited by sample size or survey design. In such cases, most constraint-based structural learning algorithms either return the set of all directed graphs that are consistent with the input data, or simply return a single, partially directed graph that only assigns directions to the edges whose d-separation implications are empirically supported. This latter type of output is a concise way of revealing all the structures that are observationally equivalent. When the directions of some edges remain ambiguous, the graph can be used to identify what additional data collection efforts are required to better reveal the underlying structure.

•***Score-based methods*** consider a number of possible BN structures and assign a score to each that measures how well it explains the observed set of data. The algorithms then typically return the single structure that maximizes the score. As the number of potential structures is super-exponential in the number of nodes, even systems with few variables have too many possible network structures to allow for an exhaustive search. Therefore, since the initial work of Chickering et al. [33] and Spirtes and Meek [34], most score-based algorithms have employed heuristic search techniques, such as hill-climbing or simulated annealing.

•There are a number of possible criteria to use for scoring BN structures. Ideally, as both the structure and parameters of the BN are typically unknown, the full marginal likelihood should be computed [35]. However, the computation of the full likelihood over both the parameter space and structure space is impractical for all but the smallest networks, requiring approximations such as the *Bayesian Information Criterion* (BIC) to be used. The BIC score (also known as the *Schwarz Information Criterion* and equivalent to the *Minimum Description Length*), can be written as:

(3) $$\mathrm{BIC}=\log\left(p\left(D\left|\hat{\theta }\right),G\right)\right)-\frac{n_{p}}{2}\log\left(N\right)$$

When a greater tolerance for complex networks is desired (e.g., in the exploratory phase of analysis), the Akaike information criterion (AIC) provides an alternative scoring function:

(4) $$\mathrm{AIC}=\log\left(p\left(D\left|\hat{\theta }\right),G\right)\right)-n_{p}$$

The AIC penalizes less harshly for the inclusion of additional edges (and their associated parameters). It is important to note that the maximum likelihood itself cannot be used as a score function, as without the inclusion of a penalty term it would always lead to selection of a completely connected network.

Cooper and Herkovits [36] propose the K2 score, which corresponds to the Bayesian posterior for the special case of a uniform prior on both the structure and parameters. The contribution of each variable to the logarithm of the K2 score can be written as:

$$\log\left(K2\left(X_{i}\right)\right)=\sum_{j=1}^{q_{i}}\left(\ln\left(\frac{\left(r_{i}-1\right)!}{\left(N_{\mathrm{ij}}+r_{i}-1\right)!}\right)+\sum_{k=1}^{r_{i}}\mathrm{In}\left(N_{\mathrm{ijk}}!\right)\right)$$

where *N*_*ijk*_ represents the number of cases in the database in which the variable *X*_*i*_ took its *k*th value (*k* = 1, 2,…, *r*_*i*_), and its set of parents was instantiated as its *j*th unique combination of values (*j* = 1, 2,…, *q*_*i*_), and $N_{\mathrm{ij}}=\sum_{k=1}^{r_{i}}N_{\mathrm{ijk}}$. The logarithm of the total K2 score is then the sum of the individual contributions. The K2 score is typically intermediate to the AIC and BIC in its penalization of network complexity.

Constraint-based methods can be more efficient than score-based approaches, especially when the number of samples is large. However, the detection of conditional independencies is sensitive to failures in hypothesis tests. Also, due to their reliance on the d-separation criterion to determine the direction of edges, they may not assign a direction to every edge. Thus, the score-based approach is generally preferred, particularly when dealing with small sample size and noisy data [37]. Hybrid algorithms have also been developed that combine the two conventional methods to maximize their advantages. Typically, they start with a constraint-based algorithm to find the skeleton of the network and then employ a score-based method to identify the best set of edge orientations.

### Common algorithmic implementations

There are, of course, multiple algorithms for implementing each of the approaches to structural learning described above. In this section, we review some of the more fundamental and popular algorithms. Many variants of each of these have also been developed in an attempt to improve efficiency and effectiveness. We do not attempt to provide a comprehensive review of these variants, nor do we attempt to provide a historical context for the algorithms described below. For these purposes, the reader is referred to the review by Daly et al. [38].

• Grow-Shrink (GS) is a constraint-based algorithm first proposed by Margaritis and Thrun [39] and based on the concept of the *Markov blanket*. The Markov blanket of a node in a BN consists of its parents, children, and its children’s other parents. These represent all the variables that can give information about the variable represented by node. The GS algorithm starts with a variable X and an empty set S. The growing phase then adds variables to S as long as they are dependent on X, conditional on the variables currently in S. In the subsequent shrinking phase, variables that are rendered independent of X, based on the current members of S, are then removed from S. Of course, the efficiency and effectiveness of this step is influenced by the order in which variables are considered. Heuristically, one could order the variables according to ascending mutual information or probability of dependence with X as computed using the *χ*^2^ statistic during the growing phase, for example [39]. The variables remaining in S after both phases then represent an estimate of the Markov blanket of X. Together with X itself, this can be represented as a fully connected, undirected network. The possible removal and direction of edges are then addressed by examining triples of variables using the d-separation criterion. Namely, spousal links between two nodes Y and Z are removed by looking for a d-separating set around Y and Z. Directions are then given to edges whenever it is found that conditioning on a middle node creates a dependency. This entire process is then repeated for every variable and the results compiled into a single network. Finally, a heuristic is used to remove any cycles that may have been introduced by previous steps [39].

• Incremental Association Markov Blanket (IAMB) is another constraint-based algorithm [40] that has similar search mechanics as GS. It also attempts to recover an estimate of the Markov blanket of each variable X through two phases of addition and removal. However, IAMB uses a dynamic heuristic function to determine the ordering of candidate variables, as opposed to the static determination of GS. This function is calculated as the mutual information between X and Y, *conditional on the current members* of the candidate Markov blanket set, S. This minimizes the size of S after the addition phase, thus reducing the number of false positives that need to be removed in the removal phase.

• Hill-Climbing (HC) is an example of a score-based algorithm [41]. The search over structures starts from either an empty, full, or possibly random graph. Alternatively, the initial graph can be chosen according to existing knowledge. The main loop then consists of attempting every possible single-edge addition, removal, or reversal relative to the current candidate network. The change that increases the score the most then becomes the next candidate. The process iterates until a change in a single-edge no longer increases the score.

• Max-Min Hill-Climbing (MMHC) is a hybrid algorithm [42] that first learns the undirected skeleton of a graph using a constraint-based algorithm called Max-Min Parents and Children (MMPC). This is followed by the application of a score-based search to orient the edges. Like the other constraint-based algorithms described above, MMPC consists of two phases when working with each variable. In the first phase, variables are sequentially connected to X by an edge according to the maximum value of their minimum association with X, where the minimum is calculated relative to all subsets of the variables currently connected to X. In the second phase, any false positives selected in the first phase are removed according to a conditional independence test. The result is an estimate of the parents and children of each node (as opposed to the full Markov blanket). These local skeletons are then directed using a greedy hill-climbing algorithm. The difference between MMHC and a standard search is that here the search is constrained to only consider adding an edge if it remained after the constraint-based phase. This gives the algorithm the advantage of reliably scaling up to thousands of variables in reasonable computational time.

## Handling missing values

When the data are complete, the modularity of BNs provided by the Markov condition (eq. 1) also facilitates the process of structural learning. This is because the likelihood function used as the basis for most scoring metrics can be decomposed into a product of terms, each of which depends only on a variable’s parents and the conditional probability parameters. This means that candidate networks generated by small changes (adding or reversing an edge) can be evaluated locally, without regard to changes made elsewhere in the network. This allows for efficient learning algorithms.

Unfortunately, when data are incomplete – as inevitably occurs with large SNP arrays and hence gene-environment-disease data – decomposition of the likelihood function is no longer possible. This makes structural learning in the presence of missing values a computational challenge. Most state-of-the-art algorithms, including the ones reviewed in the previous section, are not able to learn BNs from incomplete data. One solution might be to simply discard observations with missing values or replace them with the mean of the observations. However, in either case, statistical power is lost and the joint distribution may be distorted. Therefore, in this section we review the use of the popular expectation-maximization (EM) algorithm [43] for handling missing values in BN learning. We start with the situation of parameter learning for a known BN structure, followed by the more difficult problem of structural learning.

### Known structure

The EM algorithm is an iterative method for finding maximum likelihood estimates of parameters in the presence of missing values or latent variables. The algorithm alternates between creating a function for the *expectation* of the log-likelihood using the current parameter estimates – the E-step – and computing the parameters that *maximize* the expected log-likelihood – the M-step. The process repeats until parameter values individually converge.

Suppose, for example, we have the hypothetical binary data given in Table 1 on the variables C, E, and S1 from Figure 1. Some observations of exposure E are missing. We first use the complete data to calculate the following initial parameter estimates (simply using relative frequencies, corresponding to the maximum likelihood estimates) for the given network structure:

$$\begin{array}{l} P\left(E=1\right)=0.583\\ P\left(S1=1\right)=0.350\\ P\left(C=1|E=0,S1=0\right)=0.033\\ P\left(C=1|E=1,S1=0\right)=0.026\\ P\left(C=1|E=0,S1=1\right)=0.014\\ P\left(C=1|E=1,S1=1\right)=0.024 \end{array}$$

**Table 1:** **First five observations of dataset with some missing values represented by *NA***

**Obs.**	**E**	**S1**	**C**
1	1	1	0
2	*NA*	1	1
3	1	0	0
4	*NA*	1	0
5	0	0	1
⋮	⋮	⋮	⋮

These parameters are then used to probabilistically complete the original data set, as in Table 2. We then re-estimate the parameter values using expected frequencies and repeat the process until parameter convergence. Scores for competing network structures can then be calculated that include observations with some missing values.

**Table 2:** First five observations of probabilistically completed dataset

**Obs.**	**E**	**P(E)**	**S1**	**C**
1	1	1	1	0
2	0	0.359	1	1
	1	0.641		
3	1	1	0	0
4	0	0.417	1	0
	1	0.583		
5	0	1	0	1
⋮	⋮	⋮	⋮	⋮

### Unknown structure

As mentioned above, structural learning in the presence of incomplete data is a significantly harder problem than parameter learning. This is because the E-step of the standard EM algorithm would need to compute expected frequencies for exponentially many candidate structures before the M-step could choose the structure that maximizes the expected score. Probably the best known solution has been to embed the structural search *inside* the EM procedure [44]. Originally referred to as *model selection* EM (MS-EM) and later as *structural* EM (SEM), this algorithm alternates between searching over network structures and computing the expected frequencies for each candidate structure, on demand. Hill-climbing or other standard procedures can be used in the structural search because this step is performed as if there were complete data. A computational improvement to the standard MS-EM algorithm is to alternate between iterations that optimize the parameters for the current candidate structure and iterations that search for a new structure (AMS-EM [44]). Various generalizations of the basic SEM algorithm have since been proposed [45,46].

A fundamental problem with deterministic approaches, such as EM-based algorithms, is that they are prone to find only local maxima. While multiple random restarts may help this problem, stochastic search methods represent another solution. Myers et al. [47] present an evolutionary algorithm that evolves both the set of network structures and the values of the missing data. Myers et al. [48] describe a similar strategy using a Markov chain Monte Carlo technique.

Relatively little work has been done in developing constraint-based approaches to structural learning in the presence of missing data. Two proposed algorithms include Dash and Druzdzel’s [49] pseudo-Bayesian independence testing approach and Tian et al.’s [50] interval-based algorithm, which employs interval estimates of the joint probability of the variables obtained from possible completions of the incomplete data. These are then used to derive a point estimate for approximating the mutual information used in performing conditional independence tests. Guo et al. [51] describe a hybrid evolutionary algorithm that combines constraint-based and score-based methods for structural learning with incomplete data. To our knowledge, these constraint-based and hybrid techniques have not yet been further developed or widely applied.

## Causality

Thus far, we have implicitly assumed that the BNs being discussed obey *causal* semantics; that is, every directed edge represents an association between parent and child that can be assumed to be causal, as in Figure 1. This requires that the Markov condition of Eq. 1 is assumed to extend to the notion of causality; given the effects on a variable of its immediate causes, that variable is independent of all earlier causes. Of course, in the case of genetic data, this assumption may be indirect, as we do not necessarily suppose that SNPs themselves are causal to the disease but rather that a SNP that is in linkage disequilibrium with the measured SNP causes a functional alteration [21].

There are two more assumptions that are required in order for a BN to be interpreted as representing causality: (i) if any two variables are correlated, then it must be assumed that one is the causal parent of the other or there is a third variable causing both, and (ii) there must be no variables missing from the network that are causal parents of two or more of the variables contained in the network [18]. Therefore when any important variables of a system are left unobserved – as is often the case – it is not clear that a BN learned from data (i.e., found to be consistent with the distributions of observed values) will have a directed structure that can interpreted causally. In fact, for any single directed graph selected as “best” according to a score-based algorithm, there will generally be many other graphs that are consistent with same conditional independencies implied by the selected graph. As with the partially directed graphs emerging from constraint-based algorithms, these graphs are termed *observationally equivalent* and the set of graphs in the same *equivalence class* can be readily identified using existing algorithms [52].

Claiming that a BN represents causality is tempting because not only does a causal BN provide mechanistic insight into a system but it allows the effect of interventions (e.g., reductions in exposure, drug treatments) to be correctly predicted. Unfortunately, as stated above, the possibility of unobserved variables (often referred to as *hidden* or *latent* variables) and the existence of a typically large equivalence class usually preclude causal interpretation. On the other hand, interpreting BNs in terms of causality is not necessary for extracting meaningful information from learned structures. Schlosberg et al. [53] learn a fully directed BN involving a target phenotype and 533 SNPs using a hill climbing algorithm. Perhaps counter-intuitively, they interpret all *descendants* of the phenotype to be the causes, and compare the results to what would have been obtained had they interpreted the causes to be: (i) all members of the phenotype’s Markov blanket or (ii) only the children of the phenotype.

Sebastiani and Perls [19] provide the rationalization for the approach of Schlosberg et al. [53]: being a complex trait, one would expect the presence of disease to be modulated by a large number of SNPs. These SNPs may have modifying effects on disease status, meaning that the association between one SNP and the disease will affect the strength of association of another SNP. The consequence is that only a limited number of SNPs can be practically detected as parents of the disease in causal models that are statistically learned from data. Sebastiani and Perls suggest that this limitation is removed by the use of *diagnostic* models, in which we allow SNPs and environmental exposures to be children, rather than exclusively parents, of the disease. In this way, the ability to detect the association of each SNP with the disease is not influenced by the association of other SNPs. This type of structure also more accurately represents the data-generating mechanism of a case–control study in which subjects are chosen based on their disease status, rather than at random. In practice, because the other parents of a child of the disease also contain information on disease status, Sebastiani and Perls consider the entire Markov blanket of the disease node as potential causes. This is also the approach taken by Han et al. [54] using a constraint-based approach to structure learning. We will refer to such network representations as *non-causal*.

The probability of disease given a particular environmental exposure and genetic profile is not directly encoded in a non-causal BN, but it can be readily computed using Bayes’ theorem. This technique is used in the analyses of Sebastiani et al. [6] and Ramoni et al. [55]. Because of the counter-intuitive nature of such diagnostic models, Sebastiani and Perls [18] suggest presenting results as an undirected graph representing mutual associations, rather than causal dependencies. The undirected counterpart of a directed graph, referred to as a Markov network, is formed by connecting nodes with a common child and then removing the direction of all edges. This is equivalent to connecting each node to its Markov blanket [56].

## Example application

To exemplify the various BN structural learning algorithms and causal interpretations, we attempt to learn the relationships between a limited number of gene-environment-disease variables collected as part of a population-based case–control study of bladder cancer in New Hampshire. In addition to the presence/absence of bladder cancer, the full dataset includes over 1477 SNPs in cancer-related genes, detailed smoking assessment, gender, age, possible environmental risk factors including arsenic exposure, and selected biomarkers [57,58]. Informed consent was obtained from each participant and all procedures and study materials were approved by the Committee for the Protection of Human Subjects at Dartmouth College. In the present context, we use the BN learning method to further assess reported interactions between DNA repair genes and arsenic exposure in increasing bladder cancer risk [59]. Specifically, in assessing polymorphisms in the *XRCC3* and *ERCC2/XPD* genes using logistic regression, evidence of an increased risk of bladder cancer among those in the top arsenic exposure decile was observed for those with a variant allele of the double-strand break repair gene *XRCC3*. Therefore, we focus our analysis on 11 variables that allow us to explore the role of SNPs in the gene *XRCC3* at positions 03, 04, and 241 and *ERCC2/XPD* at positions 03, 09, and 312. Arsenic exposure is represented by toenail arsenic level as an internal biomarker, which, following Andrew et al. (2009), is dichotomized at the 90th percentile. We also include the known risk factors: gender, age (≤60 or >60), and smoking status (dichotomized as *never* and *former*/*current*). While this set of 11 variables is certainly very small for a gene association study, we believe this allows us to more clearly demonstrate the BN method.

For analysis, we use the open-source software package *bnlearn*[60] in the statistical and graphical environment *R*[61] (http://www.r-project.org). The *bnlearn* package implements all of the algorithms described in Subsection Common algorithmic implementations in addition to a number of their variants. It also supports parameter estimation for given networks, conditional probability queries, model comparison and manipulation, random data generation, and plotting.

### Complete data

We begin by comparing the Grow-Shrink(GS), Incremental Association Markov Blanket (IAMB), Hill-Climbing (HC), and Max-Min Hill-Climbing (MMHC) algorithms to a subsample of our dataset consisting of 424 controls and 226 cases without any missing values over the variables of interest.

For the constraint-based GS and IAMB algorithms and the hybrid MMHC algorithm, we used the Pearson’s *χ*^2^ test as the basis for the conditional independence tests with an alpha value (nominal type I error rate) of 0.05. Other available options in *bnlearn* include a chi-square test on the mutual information, a shrinkage estimator for the mutual information, and an experimental AIC-based independence test (see [60] for details).

For the score-based HC algorithm and the hybrid MMHC algorithm, we chose log(K2) as the scoring metric, as it is intermediate to the AIC and BIC in its penalization of complexity. Other frequentist scoring options for discrete variables in *bnlearn* include the multinomial log-likelihood, AIC, and BIC. To avoid convergence on a local maximum, we implemented 100 random restarts, each with 5 edge perturbations.

To first limit our results to fully prognostic models, we used the available ‘blacklist’ option in *bnlearn* to disallow any unreasonable casual relationships (e.g. SMOKING as a parent of AGE, non-genetic variables as parents of SNPs, CANCER as a parent of GENDER or SMOKER, SNPs as parents of AGE, TOENAIL_AS as a parent of AGE, GENDER, or SMOKER).

**Figure 2:**
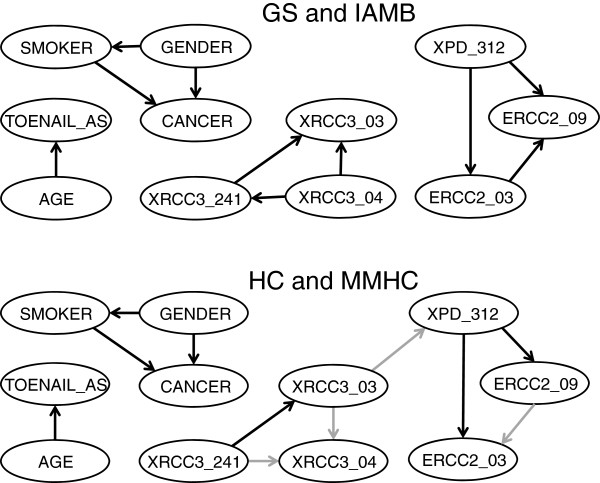
**Comparison of the BNs learned by four different algorithms.** Edges that differ between the two networks are indicated in grey in the bottom graph.

Results obtained from the four algorithms are broadly similar. In fact, the two strictly constraint-based algorithms and the two algorithms including a scoring component each returned identical structures (Figure 2). In all networks, SMOKER and GENDER are identified as parents of CANCER, and GENDER is additionally a parent of SMOKER. All networks show AGE to be a parent of TOENAIL_AS. While none of the networks show AGE as a parent of CANCER, as might be expected, this is not surprising in the present context given that controls were selected in a manner that minimized differences in age relative to cases [57].

None of the structures learned from the complete data cases shows any relation between the selected SNPs and other variables. The linkage disequilibrium groups found are the same across algorithms, with the exception of an edge between XRCC3_03 and XPD_312 in the network produced by the HC and MMHC algorithms. The directions of edges differ slightly between the strictly constraint-based algorithms and those with a scoring component, but as these are understood to only represent associations rather than genuine causal relationships this is not a serious concern.

When the networks learned by the algorithms are scored *post-hoc* using the various criteria (Table 3), it can be seen that those returned by HC and MMHC yield higher scores on all metrics except the BIC. Computationally, the constraint-based algorithms performed fewer tests than the ones with a score component, demonstrating their computational efficiency. As the computational complexity is polynomial in the number of tests, this could be a significant consideration for datasets with many more variables.

**Table 3:** Comparison of different algorithms

	**GS**	**IAMB**	**HC**	**MMHC**
Tests	259	366	750	921
Directed arcs	10		8	
Log-likelihood	-4045.78		**-4043.24**	
AIC	-4073.78		**-4072.24**	
log(K2)	-4125.07		**-4124.27**	
BIC	**-4136.46**		-4137.16	

The highest value of each score is in bold.

**Figure 3:**
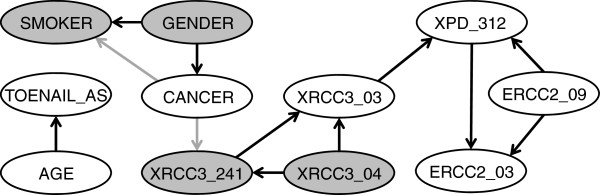
**A non-causal network.** Edges that differ relative to the causal networks are indicated in grey. Shaded nodes indicate the Markov blanket of CANCER.

We next consider a non-causal network (i.e., one in which edges do not necessarily follow causal direction) by removing from the ‘blacklist’ those edges for which CANCER is a parent and re-running the score-based HC algorithm. We choose the HC algorithm because of the results of Table 3 and because, having dropped the assumption of causality, we are interested in high scoring networks, rather than those that accurately represent causal direction. The result (Figure 3) shows three variables: GENDER, SMOKER and XRCC3_241 to have direct relations with CANCER, the first as a parent and the second two as children. Additionally, as a parent of XRCC3_241, XRCC3_04 is in the Markov blanket of CANCER. Therefore, all four of these variables should be considered further as candidate contributory causes of bladder cancer.

### Missing data

**Figure 4:**
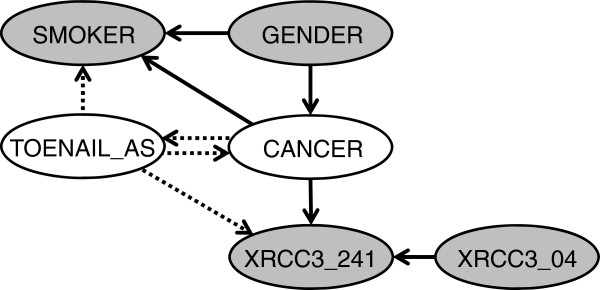
Candidate structures (represented by dotted edges) in which TOENAIL_AS would be included in the Markov blanket of CANCER.

Andrew et al. [59] found evidence of gene-environment interaction between XRCC3 241 and high toenail arsenic levels on bladder cancer risk. Thus far, our BN analysis using only the 424 controls and 226 cases with complete data has not been able to confirm this association. To investigate the possible influence of missing values, we next employed the EM algorithm to enable us to use the full dataset of 665 controls and 448 cases. (Most of the missing values occur in the variables representing SNPs.) To simplify the search process, we only consider the hypothesis that TOENAIL_AS should be included in the Markov blanket of CANCER. This could occur if this variable is either a parent of CANCER, a child of CANCER, or another parent of a child of CANCER (Figure 4). Thus, including the network representing the null hypothesis, we consider five possible structures. After application of the EM algorithm to each structure, the expected values of the log-likelihood, AIC, log(K2), and BIC scores were calculated as the basis for model comparison.

**Figure 5:**
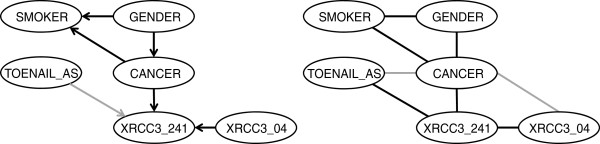
**Final structure with directed edges in which no causal interpretation is implied (left) and equivalent undirected Markov network (right).** Grey edges are new relative to earlier structures.

Results indicate that the network including TOENAIL_AS as a parent of XRCC3_241 provides the best fit to the data according to the first three scoring criteria (Table 4). Only the BIC score favors the null hypothesis of no association. The inclusion of TOENAIL_AS in the Markov blanket of CANCER is a result that is qualitatively consistent with the findings of Andrew et al. [59]. Additionally, the fact that TOENAIL_AS is a common parent of XRCC3_241, together with CANCER and XRCC3_04, indicates the presence of gene-environment interactions in determining cancer risk (Figure 5).

**Table 4:** **Expected scores for the five candidate structures shown in Figure**4**after applying the EM algorithm**

**Added edge**	**Log-Likelihood**	**AIC**	**log(K2)**	**BIC**
none	-3824.1	-3837.1	-3861.7	**-3869.7**
TOENAIL_AS → CANCER	-3824.6	-3839.6	-3865.7	-3877.2
CANCER → TOENAIL_AS	-3824.6	-3838.6	-3865.3	-3873.7
TOENAIL_AS → SMOKER	-3820.9	-3837.9	-3864.6	-3880.5
TOENAIL_AS → XRCC3_241	**-3815.8**	**-3832.8**	**-3859.6**	-3875.4

The highest scoring network according to each score is in bold.

To quantitatively explore these interactions, the risk of an individual having bladder cancer given gender, smoking status, toenail arsenic level, and XRCC3 04 and XRCC3 241 variation can be computed using Bayes’ theorem as applied to the final network in Figure 5. We used the *cpquery* function in *bnlearn* to perform the inference required to calculate the conditional probabilities of cancer. We then computed odds ratios relative to a female, non-smoker, with low toenail arsenic levels and the wildtype allele at both the 04 and 241 positions on the XRCC3 gene. The relative odds ratios of some of the 32 possible combinations (Table 5) show that, when the known risk factors gender and smoking are each present alone, they are associated with comparable increases in bladder cancer risk (see cases 6 and 7). For females without other observed risk factors, the variant allele at position 04 is associated with higher bladder cancer risk than the double wildtype form of this gene, while a double-variant form is associated with lower risk (compare cases 1, 2, 3, and 4). The association between cancer risk and arsenic exposure depends on genotype. For any combination of gender and smoking status, the risk of bladder cancer is elevated when toenail arsenic levels are high for those with a variant at position 241 and wildtype at position 04 (compare cases 3, 5, 9 and 11 and cases 10 and 13). However, for those with a wildtype at position 241 and variant at position 04, the cancer risk does not increase with arsenic level (compare cases 12 and 13). A double variant genotype is associated with lower risk of bladder cancer than single variant genotypes (compare cases 1, 3 and 4, and cases 8, 12, and 14). Such results show the potential for BNs to capture the complex role of multiple SNPs, as well as their interplay with exposure and background variables in determining disease susceptibility. Traditional logistic regression results corresponding to the relations discovered in the final BN confirm the statistical significance of the single factors, as well as the interaction between XRCC3_241 and XRCC3_04 (Table 6). The combination of high arsenic exposure and XRCC3_241 variation reveals an elevated odds ratio but an insignificant p-value of 0.14, suggesting that further study is necessary to confirm this association.

**Table 5:** Prognostic bladder cancer risk for some of the 32 possible combinations of risk factors

**Case**	**Gender**	**Smoker**	**Toenail As**	**XRCC3_241**	**XRCC3_04**	**Odds ratio**	**# of subjects**
1	female	no	low	variant	variant	0.68	13
2	female	no	low	wildtype	wildtype	1.0 (ref)	22
3	female	no	low	variant	wildtype	1.10	16
4	female	no	low	wildtype	variant	1.45	39
5	female	no	high	variant	wildtype	2.02	2
6	female	yes	low	wildtype	wildtype	2.22	27
7	male	no	low	wildtype	wildtype	2.23	20
8	male	yes	high	variant	variant	2.25	5
9	female	yes	low	variant	wildtype	2.45	24
10	male	yes	low	variant	wildtype	4.36	181
11	female	yes	high	variant	wildtype	4.48	9
12	male	yes	high	wildtype	variant	5.17	14
13	male	yes	low	wildtype	variant	5.75	95
14	male	yes	high	variant	wildtype	7.99	14

**Table 6:** Logistic regression results for associations discovered in final BN model

	**Coefficient**	**Std. error**	**P-value**	**Odds ratio**	**95% CI**
Intercept	-1.382	0.170	<0.00001	1	-
S	0.640	0.154	0.00003	1.90	(1.40, 2.56)
G	0.633	0.143	<0.00001	1.88	(1.42, 2.49)
X4	0.403	0.179	0.024	1.50	(1.05, 2.12)
A:X241	0.435	0.295	0.140	1.55	(0.87, 2.76)
X241:X4	-0.731	0.240	0.002	0.48	(0.30, 0.77)

## Discussion

While our example application is quite small in comparison to genome-wide association studies, it exemplifies some important points regarding BN structural learning. First, while the claim that a BN can represent causal relations is tempting, strictly enforcing a causal interpretation by disallowing nonsensical causal directions can limit the identification of important associations. For example, while the influence of gender and smoking on bladder cancer was clearly identified by our causal model (Figure 2), the association of cancer with the XRCC3_241 SNP was missed. This may not be surprising, given that cancer is effectively the independent variable in the process of selecting subjects for a case–control study. In the non-causal model (Figure 3), the XRCC3_241 SNP is identified as a child of CANCER (as is SMOKER), while GENDER continues to be a parent of both CANCER and SMOKER. This is consistent with the role of GENDER as a consideration in the process of selecting controls.

Interestingly, in the non-causal model, the XRCC3_04 SNP is revealed as being associated with CANCER by virtue of its membership in the Markov blanket. The V-structure that is formed at the XRCC3_241 node is a distinctive feature of BN modeling; such a structure implies that the two parents are marginally (i.e., unconditionally) independent, but become dependent when conditioned on the value of the child. A chi-squared test applied to the data confirms the fact that XRCC3_04 and CANCER are marginally independent (χ^2^ = 0.85, p = 0.36, n = 1113), but become significantly positively associated (χ^2^ = 7.75, p = 0.005, n = 558) conditional on XRCC3_241 being wildtype. A one-at-a-time search strategy (e.g. Andrew 2009) will typically miss such an association. This is an example of Simpson’s paradox, and appropriately capturing such situations in a BN allows for the accurate representation of complex relations.

In most real-world datasets, much information is lost when only complete observations are considered in statistical analysis. The EM algorithm provides a practical means for estimating model parameters without disregarding observations with missing values. In our example, this greatly increased our sample size and allowed for the discovery of toenail arsenic levels as a significant predictor of bladder cancer. TOENAIL_AS was not determined to be a parent of CANCER, but rather was found to be a parent of XRCC3_241, together with CANCER and XRCC3_04. This indicates the presence of gene-environment interactions, as reflected in the pattern of odds ratios calculated from this structure.

By focusing our attention on the role of TOENAIL_AS and its potential membership in the Markov blanket of CANCER, we were able to perform a comprehensive comparison of candidate structures after implementing the EM algorithm on each. Normally, one would be interested in comparing too many different structures to apply EM and scoring algorithms to them all. In such cases, the SEM algorithm or one of its variations would be necessary. Structural learning in the presence of missing data continues to be an active area of research.

We employed a score-based method when learning our non-causal structure and when considering TOENAIL_AS as a member of CANCER’s Markov blanket. While constraint-based methods can be more efficient, they can also be sensitive to failures in hypothesis tests. Further, if one has already given up causal claims, then a high-scoring structure is more important than a network that accurately represents causal direction. Of course, the choice of scoring metric is influential in determining which network is “best.” Because we view the process of BN structural learning as primarily an exploratory phase of data analysis that should be followed by rigorous replication studies [62], we do not see a need to rely on scoring criteria, such as the BIC, that penalize harshly for the number of edges.

Our example contained all discrete or easily discretized variables. While we believe this is generally representative of problems concerning the genetic dissection of disease, it may be that results are sensitive to the particular discretization of continuous variables. For example, based on the analysis of Andrew et al. [59], we divided toenail arsenic levels into “low” and “high” based on the 90th percentile of the observed values. There may be reasons why other thresholds would be more predictive or biologically relevant. When there is no clear basis for choosing the thresholds, other investigators have used the quartile boundaries [19]. Continuous variables could also be kept as such, in which case the conditional probability distributions in eq. (1) are represented by conditional density functions [35]. This does not necessarily present a problem for learning BN parameters for a given structure, as statistical methods – including the handling of missing data – are readily available. However, structural learning becomes significantly more difficult when variables are continuous, as the number and type of possible dependence relations and interactions becomes infinite. However, under some assumptions, such as linear relations and conditionally normal distributions, effective algorithms have been worked out [63]. Using kernel density estimators to model the conditional distribution, Hofmann and Tresp [64] were able to eliminate the reliance on normality. Further research is being conducted on this practically relevant topic [38].

We constrained ourselves here to discussing networks containing only variables for which there are some recorded data. Of course, in most cases, not all relevant aspects of a problem have been observed. Such *hidden variables* can present a problem for network structural learning, as omission of nodes effectively amounts to marginalization of the underlying joint distribution, potentially leading to complex dependencies among the remaining, observed variables. For example, in the gene-environment-disease context, data may be available on several environmental biomarkers and health outcomes, as well as a number of predisposing genetic or sociocultural factors. The relation between these is presumably mediated by the level of exposure to an environmental stressor, rendering many of the effects and predisposing factors conditionally independent. However, if the actual exposure level is not being measured, then all the observed variables will appear to be related to each other, likely in complex ways. By explicitly including a node representing a hidden variable in a network – even if there are no recorded data on that variable – the learned models are likely to be simpler and less prone to overfitting [65]. Fortunately, the structural EM algorithm can handle hidden variables analogously to how it handles missing values of otherwise observed quantities [65]. However, how to choose the number and placement of hidden variables in a BN remains an active area of research.

Despite being applied to Bayesian networks, the process of structural learning as we have described it this far has not been truly *Bayesian* in spirit. That is, we have not incorporated *prior* knowledge regarding possible structures or attempted to calculate *posterior* probabilities of model structures or features (e.g. the presence of a particular edge or the value of a particular parameter). As mentioned in Section Methodological approaches, the BIC score represents an approximation of the full marginal likelihood, which is comparable to the Bayesian posterior with a vague prior. The K2 score also represents a special case of the Bayesian posterior resulting from a uniform Dirichlet prior. A further generalization, still assuming a Dirichlet distribution but with prior knowledge able to be incorporated as an *equivalent sample size* was developed by Heckerman et al. [66] and is referred to as the Bayesian Dirichlet equivalent (BDe) score. Friedman [65] describes a version of the structural EM algorithm that directly addresses Bayesian model selection.

Even when the model selection process is Bayesian, in that it incorporates prior knowledge, typically only the single structure with the greatest posterior probability is maintained for further prediction and inference. However, when the amount of data (i.e. number of observations) is small relative to the size of the network (i.e. number of nodes or edges), there are likely to be many structures that conform with the data nearly equally well [67]. In such a situation – which is the norm when working with gene-environment-disease data with many SNPs relative to the sample size – the choice of a single “best” structure is largely arbitrary. Another data manifestation from the same population could have led to a very different final model. Zhang [68] has developed a fully Bayesian graphical method for large-scale association mapping, called BEAM3, which yields posterior probabilities of association. Yet, in most situations there are exponentially many structures that provide a “reasonable” representation of the observed data, making comprehensive investigation or communication of all “good” structures (i.e. those with a non-negligible posterior probability) impossible. For this reason, Friedman and Koller [67] propose a method for computing the Bayesian posterior of a particular structural feature, defined as the total posterior probability of all structures that contain the feature. Such features might include the presence of a particular edge between two nodes, the choice of a node’s parents, or the Markov blanket of a node. In some cases, robust assessment of such features may be more relevant to biological discovery than full articulation of (potentially fragile) network structures.

## Competing interests

The authors declare that they have no competing interests.

## Authors’ contributions

CS performed the data analysis and drafted the manuscript. AA and MK provided and summarized the data and assisted with results interpretation and writing. MB conceived of the study, provided overall direction, assisted with data analysis and results interpretation, assisted with the writing, performed final editing, and secured financial support for the study. All authors read and approved the final manuscript.
